# Structural Analysis of Viral Infectivity Factor of HIV Type 1 and Its Interaction with A3G, EloC and EloB

**DOI:** 10.1371/journal.pone.0089116

**Published:** 2014-02-26

**Authors:** Kauê Santana da Costa, Elcio Leal, Alberto Monteiro dos Santos, Anderson Henrique Lima e Lima, Cláudio Nahum Alves, Jerônimo Lameira

**Affiliations:** 1 Laboratório de Planejamento e Desenvolvimento de Fármacos, Instituto de Ciências Exatas e Naturais– ICEN e Instituto de Ciências Biológicas, Universidade Federal do Pará, Belém, Brazil; 2 Faculdade de Biotecnologia, Universidade Federal do Pará, Belém, Brazil; University of Alberta, Canada

## Abstract

**Background:**

The virion infectivity factor (Vif) is an accessory protein, which is essential for HIV replication in host cells. Vif neutralizes the antiviral host protein APOBEC3 through recruitment of the E3 ubiquitin ligase complex.

**Methodology:**

Fifty thousand Vif models were generated using the *ab initio* relax protocol of the Rosetta algorithm from sets of three- and nine-residue fragments using the fragment Monte Carlo insertion-simulated annealing strategy, which favors protein-like features, followed by an all-atom refinement. In the protocol, a constraints archive was used to define the spatial relationship between the side chains from Cys/His residues and zinc ions that formed the zinc-finger motif that is essential for Vif function. We also performed centroids analysis and structural analysis with respect to the formation of the zinc-finger, and the residue disposal in the protein binding domains. Additionally, molecular docking was used to explore details of Vif-A3G and Vif-EloBC interactions. Furthermore, molecular dynamics simulation was used to evaluate the stability of the complexes Vif-EloBC-A3G and Vif-EloC.

**Principal Findings:**

The zinc in the HCCH domain significantly alters the folding of Vif and changes the structural dynamics of the HCCH region. *Ab initio* modeling indicated that the Vif zinc-finger possibly displays tetrahedral geometry as suggested by Mehle et al. (2006). Our model also showed that the residues L146 and L149 of the BC-box motif bind to EloC by hydrophobic interactions, and the residue P162 of the PPLP motif is important to EloB binding.

**Conclusions/Significance:**

The model presented here is the first complete three-dimensional structure of the Vif. The interaction of Vif with the A3G protein and the EloBC complex is in agreement with empirical data that is currently available in the literature and could therefore provide valuable structural information for advances in rational drug design.

## Introduction

The Virion Infectivity Factor (Vif) is an accessory protein, which is essential for HIV replication. Vif activity against the APOBEC gene family depends on forming complexes with the cellular proteins ElonginB-ElonginC (EloBC), Nedd8, Cullin5 (Cul5), and RING-box 2 (Rbx2) [1]-[5]. Human APOBEC3 proteins exhibit varying degrees of inhibitory activity against retroviruses, such as HIV and SIV [6], [7], hepadnavirus HBV [8] murine leukemia virus (MLV) [9] and retrotransposons [10], [11]. However, APOBEC3G (A3G) has been shown to be a prominent member of cytidine deaminases, which displays higher retroviral activity against Vif-defective HIV-1 [6], [7]. In the absence of Vif, A3G is incorporated into budding virions, and in the next cellular infection it causes hypermutation in the viral genome by deamination of the cytosines, thus converting cytosine into uracil [12]–[14]. It is important be pointed out that Vif has many others important biological functions for HIV life cycle such as: G2 cell cycle arrest, suppression of A3G protein synthesis, inhibition of A3G packaging into the virus particles and RNA chaperone activity [15]–[20]. We would also like to mention that the structure of Vif and its interaction with APOBEC3G and other proteins of the E3 ubiquitin ligase complex have been extensively studied owing to the potential drug-oriented therapy [21].

Vif consists of several functional domains involved in APOBEC and E3 ubiquitin ligase complex recognition, but there are currently three well characterized domains [22]. The first domain is referred as SOCS-box and includes the BC-box and the Cullin-box motifs [23]. The BC-box has a high affinity for the EloBC complex [23], [24] and contains a highly conserved consensus sequence near the C-terminus region called ^144^SLQ(Y/F)LA^149^ that binds to Elogin C (EloC) by hydrophobic interactions [4], [23]. The Cullin-box contains the ^161^PPLP^164^ motif that interacts with Cul5 and C-terminal region of EloB [23], [25]. The PPLP motif is also involved in Vif multimerization and could be important for HIV-1 infectivity [26]–[28]. The second domain is referred as HCCH, which is located downstream of the BC-box, and corresponds to the residues 100–142 [2], [29], [30]. The HCCH domain is highly conserved among different Vif lentiviruses [31] and is responsible for mediating the Cul5 interaction [2], [24], [29]. This domain contains two cysteine residues (C114, C133) and two histidines (H108, H139), which are critical components of the polypeptide sequence for forming a zinc-finger motif [2], [24].

The A3G binding site is the third Vif domain. This region is essential for directing A3G ubiquitination by the E3 ubiquitin ligase complex and corresponds to a nonlinear region located at the N-terminus [32]. Two sequences appear to be important: the first is the ^14^DRMR^17^ motif and interacts with APOBEC3F (A3F) sequence (128–130), which is also located at the N-terminus [33], [34]. The other region that plays a functional role is ^40^YRHHY^44^ motif; however, this is important only for A3G interaction [35], [36]. Common binding sites of Vif to A3G and A3F include ^21^WKSLVK^26^ [37], [38], ^69^YxxL^72^ [39] and ^55^VxIPLx_4−5_LxΦx_2_YWxL^72^ [32]. Furthermore, Vif also has specific tryptophan residues at positions W5, W21, W38 and W89 that are involved in A3G and A3F binding [40].

Despite the accumulation of structural information and the molecular mechanism of action of the HIV Vif, the full-length three-dimensional structure of Vif has not been reported yet [22], [23], [41]–[44]. Recently, we have used homology modeling, molecular docking and Molecular Dynamics (MD) simulations to study different biomolecular systems [45]–[47]. In this report, we propose a complete 3D model of HIV Vif by the *ab initio* approach. The model was further analyzed using a previously resolved crystallographic structure of the BC-box of Vif. The present work also clarifies the Vif-A3G, and EloBC complex interactions by molecular docking. We also have investigated the stability of Vif-EloBC-A3G complex by molecular dynamics simulations. In addition, Vif model obtained in this study can be used as starting point to understanding the structural features of virus-cell interactions and might be used as a target for the rational design of new inhibitors of HIV-1.

## Materials and Methods

### Ancestral Sequence Inference

To avoid the use of an arbitrary sequence, we opted to use the ancestral reconstruction approach. Coding sequences of Vif were obtained from the Los Alamos HIV Databank. Then, a multiple alignment composed by subtypes (*i.e*., A1, A2, B, C, D, F1, F2, G, H, J, and K) of group M of HIV-1 was made using the MUSCLE program [48]. This alignment was further edited in order to maintain the reading frames of the Vif protein. The alignment was used to infer a maximum likelihood (ML) tree, assuming the HKY model [49], as implemented in the PhyML software [50]. This ML tree was then used to infer the ancestral Vif sequence of subtype B. This inference was performed using the model 2 (M2: selection) that allows different proportions of conserved sites (dN/dS (ω) = 0), neutral sites (ω = 1) and an additional class of sites with its ω ratio (which can be >1) estimated directly from the data, this analysis was done by the CODEML program from the PAML v. 3.14 package. [51].

### Vif Modeling

The ancestor sequence of the subtype B of HIV-1 was modeled using the AbinitioRelax modeling protocol in the Rosetta software package (version 3.2). The Rosetta algorithm uses a Monte Carlo strategy to assemble short fragments of three- and nine-residues of known protein structures followed by gradient-based refinement with respect to all backbone and side chain torsional angles in a detailed all-atom force field to obtain compact structures [52], [53]. To model the zinc-finger motif that is essential for Vif function, we used constraints that defined the spatial relationship between the side chains of Cys/His residues and zinc ions. This archive contains six internal coordinate parameters for each amino acid: bond angles, torsion angles, bond length and geometry of the zinc-finger motif, as previously defined [54]. The zinc-finger was modeled assuming tetrahedral geometry with four atoms positioned at the vertices and the zinc ion at the center considering a distance of 2.20 Å; virtual atoms were included in the constraints archive to enforce tetrahedral coordination arrangements. Two fragment libraries of nine and three residues in length were generated on the Robetta server (http://robetta.bakerlab.org/) using the FASTA format sequence specifying the zinc-finger residues positions. Fifty thousand models were generated, and then 1% (500) of the structures with the lowest free energy was selected from the total. We also used the MaxCluster program to perform an alternative centroid analysis [55]. Then, we carried out structural analysis with respect to zinc-finger formation (C115, C134, H109, H140), and the residues disposal in the binding domains. After model selection, the Rosetta performed loop-refinement, with the zinc-finger structure incorporated.

### A3G Modeling

The coding sequence of the N-terminal region of human APOBEC3G (residues 1–194) was obtained from GenBank (www.ncbi.nlm.nih.gov/genbank/) under the accession number: NP_068594. Three templates were selected from the RCSB Protein Data Bank (http://www.rcsb.org): the C-CDA of human A3G (PDB ID code: 3IQS and 2KBO) both with identity 38.92%, and the human APOBEC2 (PDB ID code: 2NYT) with 30.77% identity. Then we performed the multiple sequences alignment that was used for modeling performed in the Modeller software [56]. One hundred structures were generated and selected by the Ramachandran plot and the Root Mean Square Deviation (RMSD). After selecting the model, we performed loop-refinement using Modeller and the model was then submitted on the ModRefiner [57] server to obtain atomic-level energy minimization and a reliable stereochemistry quality. Finally, the ion Zn^2+^ was added to the structure using the atomic coordinates of homologous APOBEC2 to form the zinc-finger motif.

### Molecular Docking and Complex Assembly

We performed a local docking between the Vif, EloB, EloC and A3G and the binding sites were selected according to the experimental information of literature. The BC-box motif of Vif was docked against EloC obtained from PDB server (3DCG, chain D) and the C-terminal region of EloB protein (2C9W, chain B) was docked against Vif -EloC complex. Finally, the ^40^YHHRY^44^ motif located at the N-terminus of Vif was docked against the N-terminal region of the modeled A3G. One thousand structures were obtained in each molecular docking. The local docking was carried out in the RosettaDock program using the flexible docking protocol with 15 cycles of Monte Carlo minimization. This protocol applies the Rosetta algorithm that simultaneously optimizes the side-chain conformations and rigid body positions of the two docked proteins, followed by a rigid-body Monte Carlo Minimization (MCM) strategy.

### Molecular Dynamics and Model Selection

Among the structures obtained by the Rosetta score, ten models of Vif-EloBC-A3G complex, were selected to employ a short molecular dynamics simulations of 5 ns and re-score them by free-energy calculations using the Solvated Interaction Energy (SIE) method available in SIETRAJ program [58], [59]. Whereas the model has been selected, the following protocol was employed in order to check the stability of the Vif protein inside and outside the complex, the tetrahedral coordination stability of zinc-finger motif and the interactions between residues of Vif-EloBC-A3G complex.

We carried out molecular dynamics simulations using AMBER 12 software package [60]. The Cationic Dummy Atom (CaDA) approach [61], [62] and the AMBER FF99SB force field were employed to treat the zinc-finger motif and the rest of the system, respectively. The protein was solvated in a truncated octahedron TIP3P water box. The distance between the wall of the box and the closest atom of the solute was 12.0 Å, and an explicit solvent was used and the closest distance between the solute and solvent atoms was 0.8 Å. The center of the mass in Vif molecular dynamic simulations corresponds to the protein itself and the Vif-A3G and Vif-EloC to the complex. Counterions (Cl^−^) were added to maintain the electro-neutrality of the system: 12 Cl^−^ in Vif protein, 10 Cl^−^ in Vif-EloC complex, and 14 Cl^−^ in Vif-EloBC-A3G complex. First, all hydrogen atoms were relaxed through 2000 steps of steepest descent. Next, the position of water molecules was relaxed using 2000 steps of steepest descent, followed by 3000 steps of conjugate gradient. Finally, the whole system was optimized through 3000 steps of steepest descent plus 5000 steps of conjugate gradients. Afterwards, we started the thermalization of the system running 50-ps molecular dynamics (MD) simulations to increase the temperature up to 300 K. Subsequently, a 10 ns MD simulation was carried out at 300 K. In addition, SHAKE was used to restrain the hydrogen positions at their equilibrium distances, which allowed the use of an integration time step of 2 fs. Finally, a cutoff of 11 Å was used for no bonded interactions.

Free energy calculations were employed in order to investigate the binding affinity between these proteins. Then, ptraj program (included in AMBER 12 software package) was employed to select the MD snapshots, which were used to calculate the average of the free energies results obtained from each snapshot by SIETRAJ program. The SIE method is a method that shares elements some features with the Linear interaction energy (LIE) and Molecular Mechanic/Poisson-Boltzmann Surface Area (MMPBSA) methods [58], [59]. The last 5 ns of MD trajectory, which represents 500 snapshots, were used to compute the binding free energy of Vif in complex with the cellular proteins EloB, EloC and A3G.

Additionally, mutations of the better model were employed in order to verify the effects in total binging free energies and study the function of the some residues in the interactions of these proteins.

### Models Validation and Analysis

The A3G model selected after molecular dynamics was validated considering the stereochemistry quality in PROCHECK tool (Ramachandran plot) [63] and the system stability by ANOLEA [64] and Qmean [65] available in Swiss-Model Server [66]. The model of Vif-EloBC-A3G structure was visualized using PyMOL software [67] (http://www.pymol.org).

## Results and Discussion

### Comparison with Previous Vif Models

**Figure 1:**
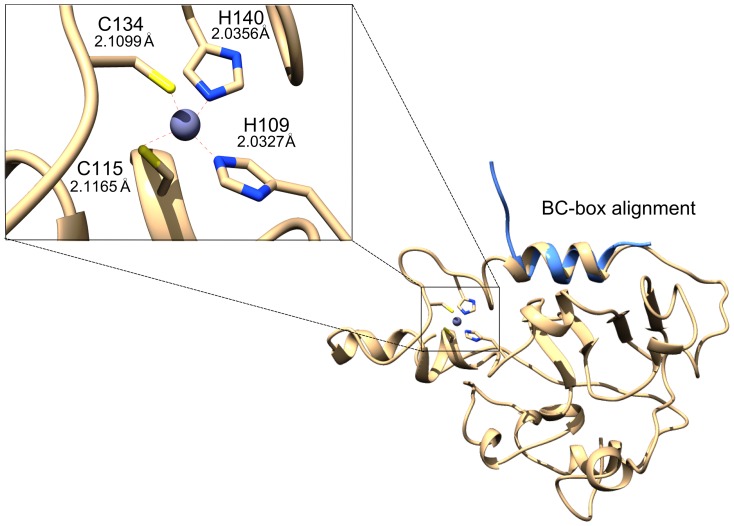
Zinc-finger motif of Vif showing tetrahedral coordination between residues C115, C134, H140 and H109 and average bond length between the atoms from Cys and His residues and the zinc ion computed after 10 ns molecular dynamics and the crystallographic structure of BC-box motif in blue (PDB ID code: 3DCG, chain E) aligned with the Vif model showed a satisfactory structures superposition.

**Figure 2:**
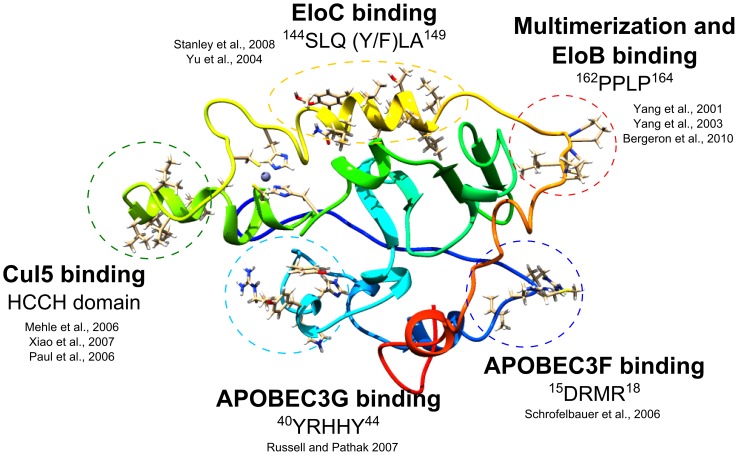
Vif theoretical structure showing the binding sites to the proteins of E3 ubiquitin ligase complex and to the A3G and A3F. The PPLP motif is located away from the hydrophobic region of HCCH domain and the BC-box exhibit the SLQ(Y/F)LA motif.

In this study, we have proposed a computational model of the Vif of HIV-1 (Fig. 1 and 2), which was based on structures with lower all-atom energy and low-resolution using the Rosetta standard protocol [52], [53]. In addition, we have used four virtual atoms in order to define the spatial coordination of the Vif zinc-finger motif [54]. After modeling, we also selected models with respect to the location of residues in the binding domains of E3 ubiquitin ligase complex proteins (EloB, EloC and Cul5) and A3G/A3F. Previously, the isopenicillin N synthase sequence from *Aspergillus nidulans* (PDB ID: 1BK0) was used as a template for modeling the structure of Vif [68]. It is important to note that the isopenicillin N synthase of *A. nidulans* has a low degree of similarity (24.67%) and a poorly established evolutionary relatedness with the Vif of HIV. This model also shows no structural correspondence with the BC-box motif of Vif, because it displays a loop conformation when experimental data shown α-helix conformation. In another study, homology and *threading* approaches were used to model the Vif. In this case, the C-terminus domain (residues142–177) of Vif was modeled using the SOCS-box sequence from VHL and the N-terminus domain was modeled by using the NarL protein from *Escherichia coli*. In that model, a sequence of 15 residues (178–192) of the C-terminus region was removed [69]. None of these models, however, incorporated data of the HCCH domain that is crucial for Cul5 interaction and for Vif folding. In addition, these models were proposed before empirical evidences, based on X-ray diffraction, showing the recruitment of the E3 ubiquitin ligase complex by the Vif BC-box motif [23].

### The Zinc-Finger Motif Alters the HCCH Domain Conformation and Exposes a Hydrophobic Region

Zinc-fingers are motifs formed by short polypeptide straps in which zinc has an important role contributing to the structural stability of the domain [70]. These motifs have different structural conformations and zinc proteins perform a wide range of functions in intracellular processes, such as signaling proteins, transcription factors and protein transport and storage. Typically, zinc binders include the side chains of cysteine, histidine, glutamate and aspartate and water molecules; a common feature of these motifs is flexibility, as they can adopt different geometries including tetrahedral, penta- and hexa-orthogonal [70], [71]. It has been shown that 82% of the zinc binding residues are cysteine and histidine, which are usually arranged in a tetrahedral geometry [72]. Consequently, we assumed that the Vif zinc-finger motif is arranged in a tetrahedral coordination in Vif modeling.

The Figure 1 shows the structure of the Vif protein, the zinc-finger of our model (enlarged panel in the Figure 1) resembles to a subtype of TAZ2 domain-like zinc finger fold group proposed by Krishna et al. (2003) [70]. However, the spacing between Cys and His residues suggests the arrangement H-X_5_-C-X_17–18_C-X_3–5_-H. These features agree with the previous suggestion that the arrangement between Cys/His residues is stable and seems to form a structure that is found only in Vif when compared with other classes of zinc-finger motifs [30]. Furthermore, the model is also in agreement with the observation that zinc binding destabilizes the HCCH domain and generates an α-helix and loop conformation [73]. These results are also in accordance with the previous secondary structure prediction obtained by Xiao et al. (2006) [30].

By assuming the tetrahedrical geometry coordination, our model exposed the side chains of the hydrophobic residues A120, I121, A124, I125, L126, I129 and V130 in the HCCH domain (Figure S1), thus demonstrating a favorable region to Cul5 binding, as previously suggested [2], [29]. Several studies have suggested that the HCCH domain conformation and Cul5 binding affinity is intimately related to the presence of a zinc-finger motif [8], [24], [31], [41]. The importance of this motif for the correct functioning of Vif justifies the low variability position of Cys and His residues found among different lentiviruses. Our model was compared with the crystallographic structure of the BC-box motif of Vif determined by Stanley et al. (2008) (2.40 Å resolutions; PDB ID: 3DCG, chain E) and the modeled BC-box juxtaposed with the crystallographic structure showed a satisfactory RMSD value of 0.904 Å (Fig. 1). A hydrophobic region in the BC-box motif (i.e., V143, L146, L149, L151 and A153) was present in the modeled Vif structure and in the crystallographic structure (Figure S1). This is consistent with the predicted hydrophobic interaction between Vif and EloC [4], [23]. Interestingly, the Cullin-box modeled by Stanley et al. (2008) presented a distinct structure compared with that of our model. These authors did not assume the zinc-finger during their homology modeling. Therefore, it is likely that lack of a structured zinc-finger may change the whole structure of Vif mainly regions in the vicinity of HCCH motif. Furthermore, Reingewertz et al. (2009) demonstrated that the Vif C-terminal domain in the unbound state to the E3 ubiquitin ligase complex proteins is in an unstructured form and thus can change its loop conformation when binding to EloB.

The function of the PPLP motif to recruit the E3 ubiquitin ligase complex has been demonstrated in distinct studies [24], [25]. The PPLP motif has therefore been suggested as responsible for Vif multimerization [3], [4], although this phenomenon is not involved in the assembly of the E3 ubiquitin ligase complex [25]. In our model, the disposition and the location of residues in the Cullin-box reinforces previous results showing that the PPLP motif interacts with EloB in the N-terminal region [25]. The binding between the PPLP motif and Cul5 implies in the occurrence of overlapping regions with the EloBC structure. The PPLP motif in our model is located away from the hydrophobic HCCH domain (Fig. 2). For this reason, we postulate that the mechanism of E3 ubiquitin ligase complex recruitment occurs exclusively in the HCCH domain. Interestingly, different studies have shown that the HCCH domain exhibits high binding affinity to Cul5, especially the region containing the zinc-finger motif [24], [29]–[31]. On the other hand, the Cullin-box interacts weakly with Cul5, and therefore its function is dispensable for the recruitment of the E3 ubiquitin ligase complex [24].

Auclair et al., (2007) showed that upon oligomerization the Vif C-terminal becomes highly ordered. They also demonstrated that Vif is in a dynamic equilibrium to form dimers and trimers while uncomplexed structures are transient forms [74]. The study indicated that the residues of C-terminal region E134, K141, K158, K160, K171, and K176 are involved in cross-linking during oligomerization. These residues (corresponding to the residues E135, K42, K159, K161, K169 and K177 in our model), are exposed in our model of the Vif structure.

### A3G Validation

The stereochemical quality of the proposed homology model of the N-terminal region of A3G was evaluated using the PROCHECK tool. The Ramachandran plot showed 92.6% (163 AA) residues in highly favorable regions (the core), 6.2% (11 AA) in additionally allowed, and 1.1% (2 AA) in generously allowed regions (Fig. S2). The model of A3G exhibits an excellent quality stereochemistry, and showed a good ANOLEA and Qmean energy score profile, thus, indicating reliability of the structure prediction (Fig. S3). The choice of models 3IQS and 2KBO is justified by the high similarity between the N- and C-terminal domains of the APOBEC proteins.

### Molecular Docking

**Figure 3:**
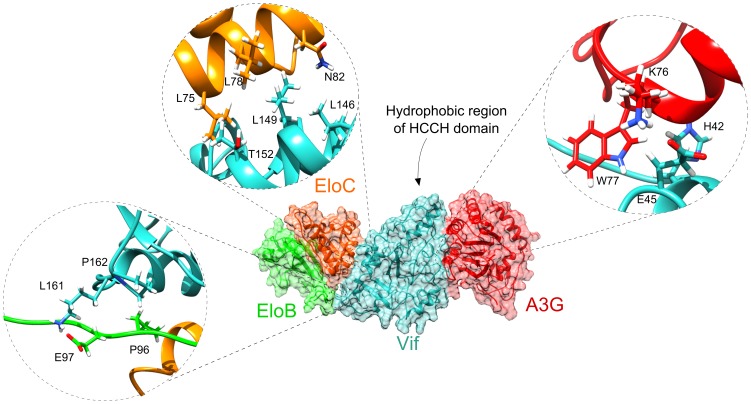
Assembled complex of Vif-EloBC-A3G N-CDA. Panels show interactions of Vif with A3G, EloC, EloB and (in the clockwise direction). In the first panel, the residues L146 and L149 from Vif BC-box are involved in EloC interaction (Vif is in cyan and EloC in orange). The second panel shows that in the N-terminal region, Vif interact via hydrogen bonding with A3G (Vif is in cyan and A3G in red). Last panel shows residues L161 and P162 from Vif Cullin-box interacting by hydrophobic interaction with the residue E97 and P96 from the C-terminal end of EloB (Vif is in cyan and EloB in green).

The molecular docking results between Vif model and EloC showed that the affinity between these proteins occurs owing to the hydrophobic interactions by residues L146, L149 and T152 from the SOCS-box and N82, L78 and L75 residues from EloC, respectively (Fig. 3). These hydrophobic interactions may explain why experimental mutations at position L145 in the SOCS-box (corresponding to L146 in the our model), drastically alters the binding affinity between Vif and EloC [4]. This result is also in agreement with the crystallographic structure obtained by Stanley et al. (2008) [23].

In the theoretical model of complex, the residue P96 of C-terminal end of EloB interacts with the residues P162 located at C-terminal region of Vif via hydrophobic interaction (Fig. 3) These results reinforce the importance of PPLP motif to the complex binding. Regarding Vif and A3G interactions, some studies have suggested that serine/threonine phosphorylation is necessary for Vif function *in vivo* [75], [76]. However, Kopietz et al. (2012) demonstrated that phosphorylation is not involved in Vif-A3G interaction [77]. Results of the molecular docking between Vif and A3G showed that the residue H42 of the ^40^YRHHY^44^ motif interact with the residues F71 of A3G via hydrogen bonding (Fig. 3). Interesting the residue E45 located adjacent to the^40^YRHHY^44^ motif also showed interaction with the residue K76 of A3G. Our data is in accordance with results reported by mutational studies, which demonstrated a requirement for the Vif motif ^40^YRHHY^44^ to interact with A3G [29], [36].

### Binding Energy of Vif-EloBC-A3G Complex and Mutation Analysis

The average binding energy (kcal/mol) obtained for each Vif model (Table S1) indicated one with the lowest binding energy that was used as the final model to perform further mutational analysis. The binding free energy of this Vif model complexed with EloB/EloC was -16.2 kcal/mol, which is agreement with the fragment value of −12.9 kca/mol determined by Wolfe et al. (2010) through isothermal calorimetric titration [24]. On the other hand, these authors also found that the C-terminal region of Vif binds EloB/EloC with subnanomolar affinity and mutations L145A (L146A in our model) and L148A (L149A in our model) drastically reduce the affinity between Vif and EloB/EloC. In addition, the SOCS-box domain showed hydrophobic interactions with the EloBC and the isolated binding energy for this complex for the model (−9.4±0.4 kcal/mol) was consistent with previous studies for this interaction where the SOCS-EloBC complex was analyzed [78]. The mutations: R41A, H42A, E45A, L146A, L149A, T152A, K161A and P162A were performed in the final model of Vif. These residues were selected for alanine substitutions since they were previously associated with important functions during Vif activity and also in the assemblage with the E3 ubiquitin ligase complex. Then free energies were calculated individually for each mutant in complex with the EloBC-A3G. Due to the high hydrophobic characteristic of the Vif-EloBC interface, mutations in the most of the residues of Vif had minor effect on the binding energy (Figure S4). However, mutation of the residues P162 and L149 on Vif, which are important for, respectively, EloB and EloC binding on the model showed great contribution for the free energy and their importance for complex stability were previously reported by different authors. The alanine scanning study for the main residues of the Vif-A3G interface showed reasonable effects (about 1kcal/mol) for the residues E45 and H42 of Vif, in our model, because these residues are involved in hydrogen-bond interactions with A3G (Figure S4).

### Dynamics and Flexibility of Vif Structure

**Figure 4:**
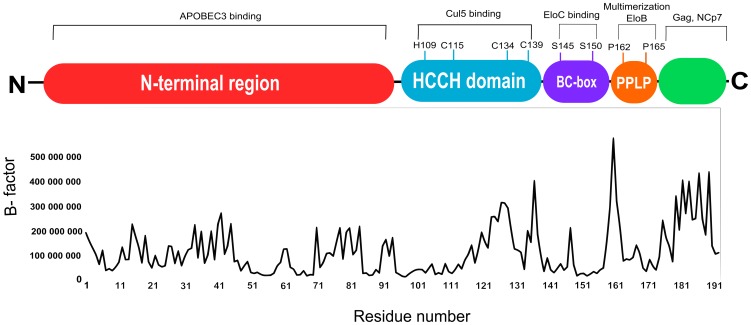
The average atomic B-factor per residue is plotted as function of the residue number and was obtained after 10ns of molecular dynamics simulation. These figure information have been adapted from Wissing et al., [87] to the Vif sequence modeled.

**Figure 5:**
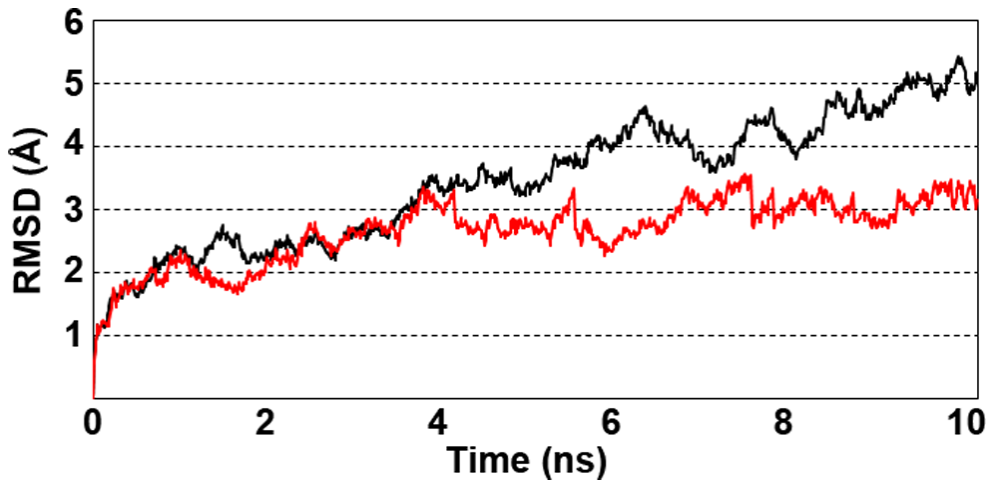
Root-mean-square deviation (RMSD) values of the Vif backbone in unbound state (black line) and its structure interacting with the EloBC-A3G complex (red line). In both cases the values were calculated over the 10

Vif has been highlighted as a high flexible protein and its C-terminal domain is natively unfolded in the unbound state [41], [79]. To explore the interaction between residues of Vif-EloBC-A3G complex, we performed 5 ns of MD simulations at 300 K for Vif and for the complex Vif-EloBC-A3G. The starting point was taken from the Vif *ab initio* model and Vif-EloBC-A3G complex obtained by molecular docking respectively. To measure the protein flexibility, we have analyzed the B-factor graph of Vif structure and compared the Root-mean-square deviation (RMSD) graph of Vif structure in unbounded state and in the complex with EloB, EloC and A3G. The B-factor shows the mobility of atoms over time, i.e., how atom deviates in relation to its middle position during the molecular dynamics. High values of B-factor indicate therefore high mobility of single atoms and side chains [80]. The B-factor averaged during the molecular dynamics simulations show that the C-terminal region seems to be highly flexible, in particular the region comprising the intervals between 120–139, 159–165 and 173–193. The first residue segment includes HCCH domain and is essential for Cul5 binding, the second, corresponds to the multimerization site and EloB binding; the third comprises different functional sites involved in the recognition of proteins Gag, NCp7 and the cell membrane proteins. The average atomic B-factor per residue was plotted as function of the residue number (Fig. 4). This high flexibility of the Vif structure can be explained by presence of many loops in the C-terminus. It is important to note, that the E3 ubiquitin ligase complex was not fully analyzed here and different components of this complex play essential role in the stability of Vif. For instance, analyzing the Vif-EloC complex, the EloC moves away from the binding site during molecular dynamics simulations, which suggest that Vif-EloC complex is not stable. On the other hand, Vif-EloBC-A3G complex seems to be stable during MD simulation. The RMSD graph of Vif interacting with the EloBC-A3G complex tend to reach the plateau so, this structure seems to be more stable than Vif in unbounded state (Fig. 5). It is important to clarify that we have used a relative short time of MD simulations just to illustrate that Vif structure is more stable when it is bounded. The stability of the Vif-EloBC-A3G complex is in agreement with empirical data showing that EloB is need to stabilize Vif binding [25], [79], [80].

It should be noted that the stability of the Vif structure is probably dependent on protein binding of the E3 ubiquitin ligase complex and to the APOBEC3 proteins. Indeed, the results of our study correlate with other studies showing that the C-terminal portion of the Vif, in which the binding sites of EloBC and Cul5 are found, are poorly preserved, exhibiting different conformations in solution [41], [79]. Bergeron et al. (2010) showed, in addition, that the BC-box motif in the absence of the EloBC complex is presented without the typical conformation of an α-helix [25]. The Vif flexibility and heterogeneity suggests, therefore, that the molecular docking with E3 ubiquitin ligase complex proteins and structural analysis with respect to the residue disposal in the binding domains was useful to determining the three dimensional structure of Vif.

Although this study has focused on the binding domains of the APOBEC3/E3 ubiquitin ligase complex, Vif displays other biological functions. For instance, Vif encapsidation requires the interaction with the genomic RNA of HIV-1 and the nucleocapsid domain of Gag [15], [81]–[84]. Other studies have showed also that Vif could bind in other viral components such as tRNA^Lys3^ [82], [85] and reverse transcriptase [86]. Recently, Zhou et al. (2012) identified that core-binding factor-beta (CBFβ), a cellular protein, interacts directly with Vif forming a stable complex [5]. The structure acquired by Vif is highly dependent on the binding to the APOBEC3/E3 ubiquitin ligase complex.

## Conclusion

One of the most important finding of present work is that the zinc is able to significantly modify Vif folding, possibly not only with regard to the tertiary structure, but also to the protein secondary structure and the structural dynamics of the HCCH region [41]. We would also like to point out that our Vif model present many residues in acceptable geometrical arrangements that can explain the Vif protein function and its interactions with A3G, EloC and EloB. Notably, in our model the Cys and His residues of the zinc-finger motif conserved a tetrahedral coordination with the zinc ion during 10 ns of MD simulations, which is consistent with the assumption proposed by Mehle et al. (2006) that the tetrahedral coordination of the zinc-finger Vif fulfills the structural and the functional requirements of the activity of this viral protein.

The model presented here is consistent with distinct empirical data of Vif function and interactions with other proteins that are currently available in the literature. Then interaction between Vif and viral components or conserved cellular proteins could impose constraints to amino acid substitutions in this viral protein. Indeed, Vif has many amino acids under epistasis, as has been revealed by the presence of co-evolving residues in this protein of HIV-1 [42]. The proposed model may be useful for the rational design of new drugs aimed at blocking the degradation of A3G/A3F induced by Vif, as well as for the elucidation of its molecular mechanism of action.

## Supporting Information

Figure S1 Two highly hydrophobic region were found in the Vif model: one is located at HCCH domain and correspond to the residues A120, I121, A124, I125, L126, I129 and V130 and the other in BC-box motif represented by residues V143, L146, L149, L151 and A153.
(TIF)

Figure S2 Ramachandran plot of the theoretical structure of A3G homology modeled.
(TIF)

Figure S3 Qmean and ANOLEA energy profile of A3G structure.
(TIF)

Figure S4 Binding energy (kcal/mol) obtained for each residue mutation of Vif complexed to EloBC-A3G N-CDA. Residues represented in blue are located in A3G, residues in green are located in EloC surface and residues in red are located in EloB surface.
(TIF)

Table S1 Average binding energy (kcal/mol) and Rosetta energy obtained for each residue mutation of Vif complexed to EloBC-A3G N-CDA. Residues represented in blue are located in A3G, residues in green are located in EloC surface and residues in red are located in EloB surface.
(DOC)
