# Correction: Structural Analysis of Viral Infectivity Factor of HIV Type 1 and Its Interaction with A3G, EloC and EloB

**DOI:** 10.1371/journal.pone.0099470

**Published:** 2014-06-02

**Authors:** The PLOS ONE Staff

There is an error in the legend for Table S1. Please view the correct legend and associated Table S1 here:

## Supporting Information

Table S1 **Average binding energy (kcal/mol) and Rosetta energy obtained for each residue mutation of Vif complexed to EloBC-A3G N-CDA.**
(DOC)
