# Supplementary figures and images for: Structural Analysis of Viral Infectivity Factor of HIV Type 1 and Its Interaction with A3G, EloC and EloB
Source: PLoS One. 2014 Feb 26;9(2):e89116. doi: 10.1371/journal.pone.0089116 (PMC3935857; doi:10.1371/journal.pone.0089116)

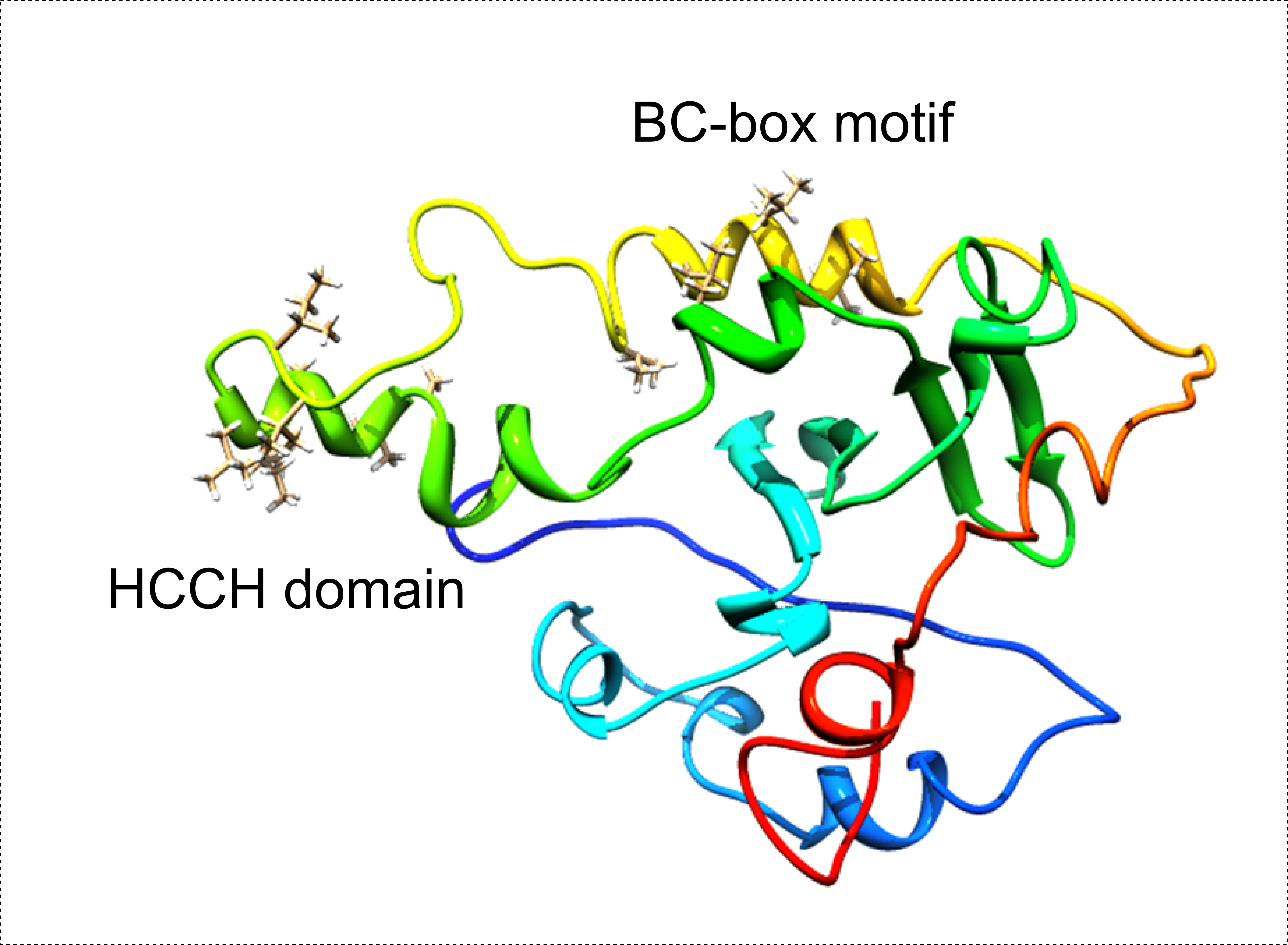

Supplement: Figure S1 — Two highly hydrophobic region were found in the Vif model: one is located at HCCH domain and correspond to the residues A120, I121, A124, I125, L126, I129 and V130 and the other in BC-box motif represented by residues V143, L146, L149, L151 and A153. (TIF) [file pone.0089116.s001.tif]

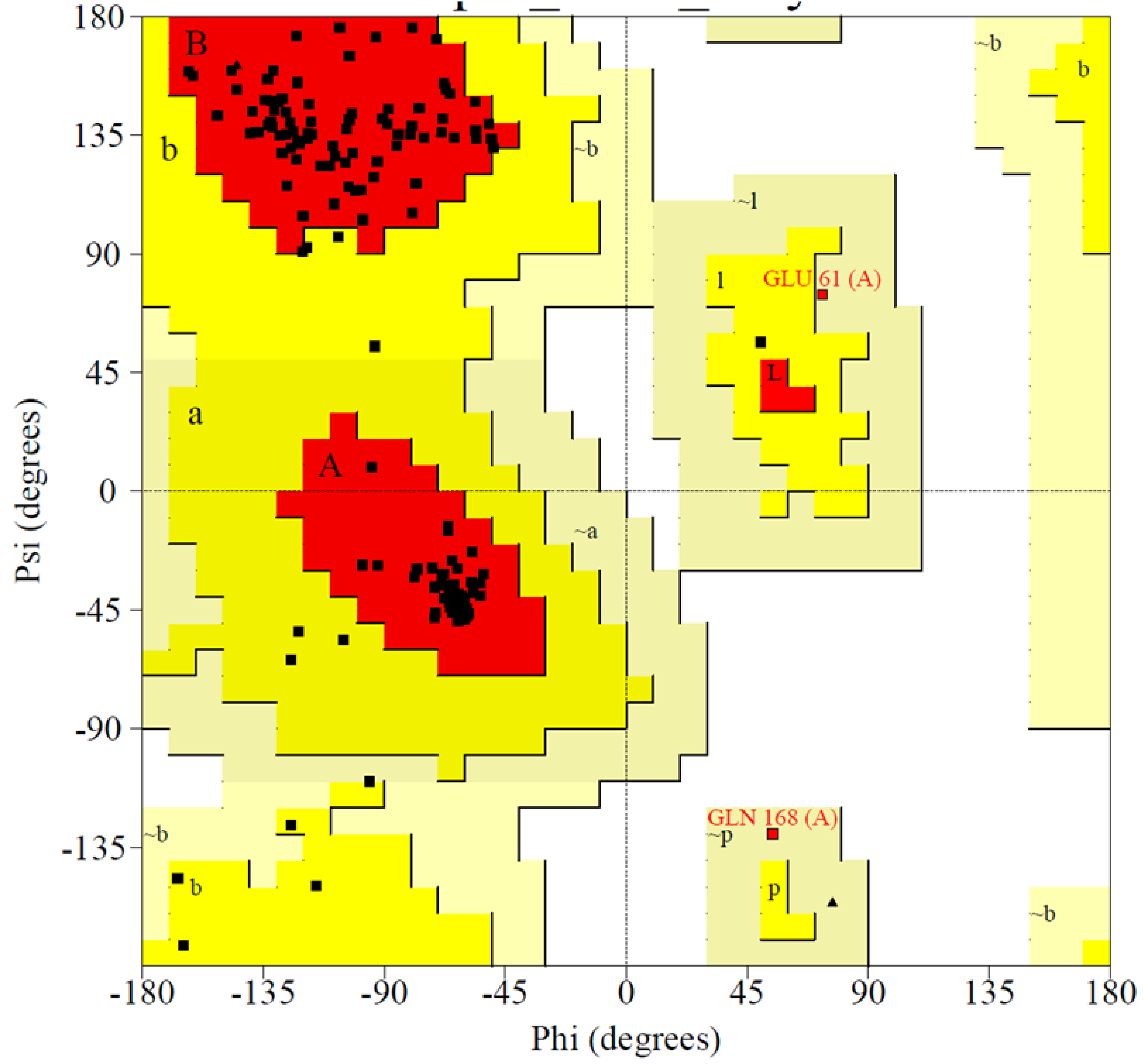

Supplement: Figure S2 — Ramachandran plot of the theoretical structure of A3G homology modeled. (TIF) [file pone.0089116.s002.tif]

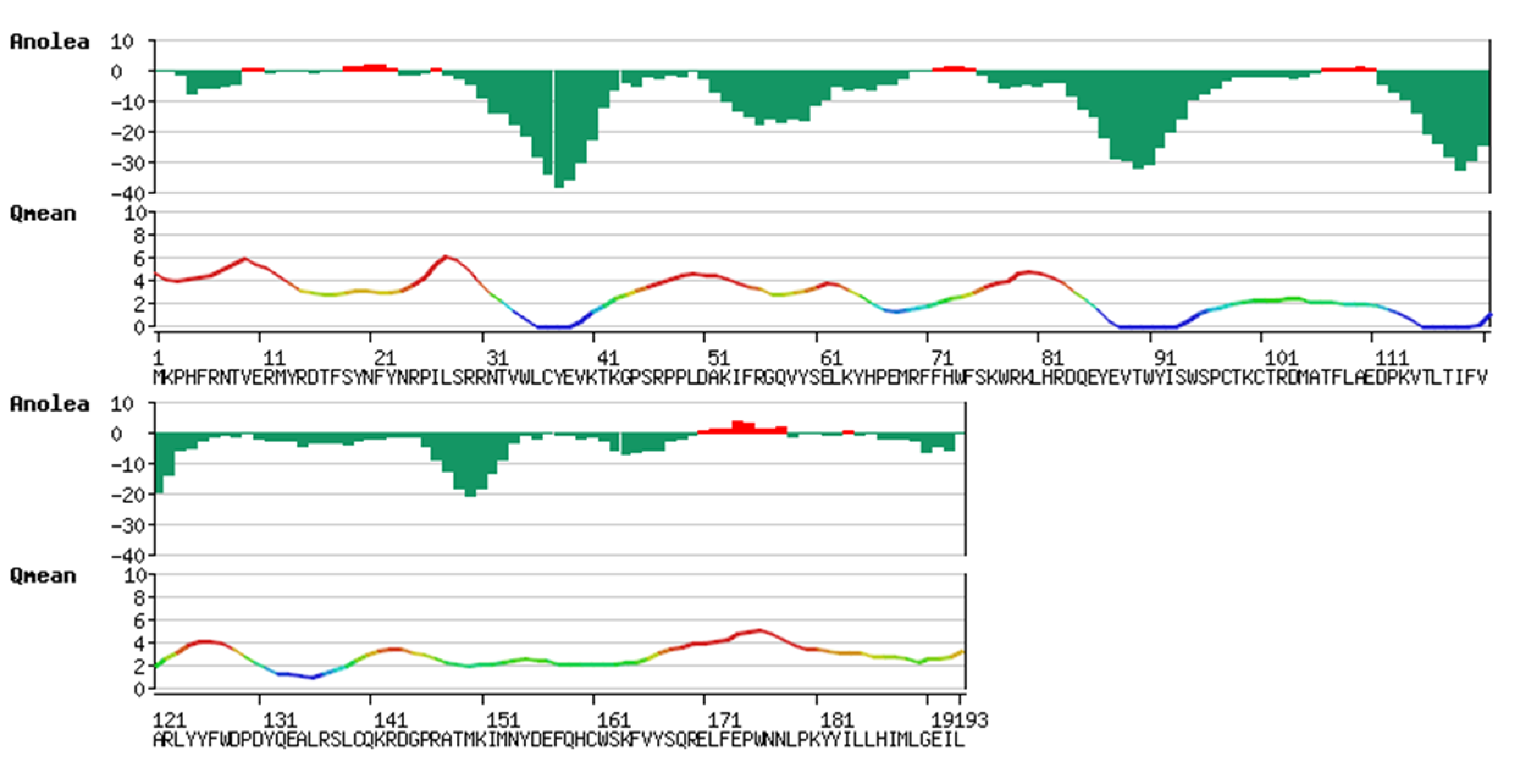

Supplement: Figure S3 — Qmean and ANOLEA energy profile of A3G structure. (TIF) [file pone.0089116.s003.tif]

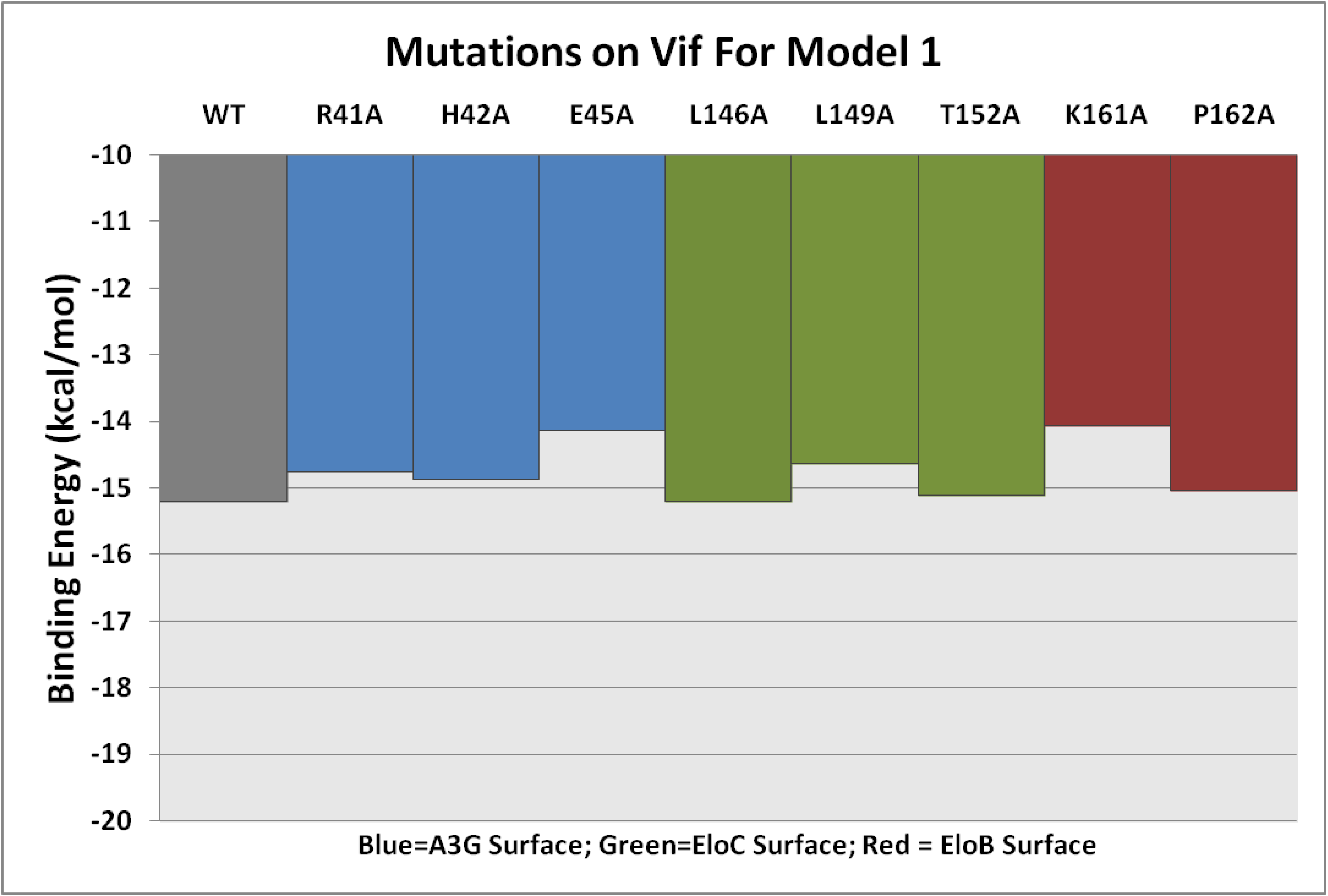

Supplement: Figure S4 — Binding energy (kcal/mol) obtained for each residue mutation of Vif complexed to EloBC-A3G N-CDA. Residues represented in blue are located in A3G, residues in green are located in EloC surface and residues in red are located in EloB surface. (TIF) [file pone.0089116.s004.tif]
