# Supplementary material for: Structural Analysis of Viral Infectivity Factor of HIV Type 1 and Its Interaction with A3G, EloC and EloB
Source: PLoS One. 2014 Feb 26;9(2):e89116. doi: 10.1371/journal.pone.0089116 (PMC3935857; doi:10.1371/journal.pone.0089116)
Supplement: Table S1 — Average binding energy (kcal/mol) and Rosetta energy obtained for each residue mutation of Vif complexed to EloBC-A3G N-CDA. Residues represented in blue are located in A3G, residues in green are located in EloC surface and residues in red are located in EloB surface. (DOC) [file pone.0089116.s005.doc]

Table S1- Average binding energy (kcal/mol) and Rosetta energy obtained for each residue mutation of Vif complexed to EloBC-A3G N-CDA.

| **Rosetta Rank** | **Rosetta Score** | **Binding energy (kcal/mol)** |
| --- | --- | --- |
| 1 | ‐519.83 | ‐16.30 |
| 2 | ‐518.817 | ‐14.38 |
| 3 | ‐517.911 | ‐15.55 |
| 4 | ‐517.866 | ‐11.6 |
| 5 | ‐517.706 | ‐13.92 |
| 6 | ‐517.624 | ‐12.32 |
| 7 | ‐517.542 | ‐12.92 |
| 8 | ‐517.453 | ‐14.38 |
| 9 | ‐517.367 | ‐14.11 |
| 10 | ‐517.286 | ‐9.98 |
